# Seed Development and Protein Accumulation Patterns in Faba Bean (*Vicia faba*, L.)

**DOI:** 10.1021/acs.jafc.2c02061

**Published:** 2022-07-21

**Authors:** Ahmed O. Warsame, Nicholas Michael, Donal M. O’Sullivan, Paola Tosi

**Affiliations:** † School of Agriculture, Policy and Development, University of Reading, Reading RG6 6AH, U.K.; ‡ School of Chemistry, Food and Pharmacy, University of Reading, Reading RG6 6AH, U.K.

**Keywords:** faba bean, seed development, protein accumulation patterns, legumin, vicilin

## Abstract

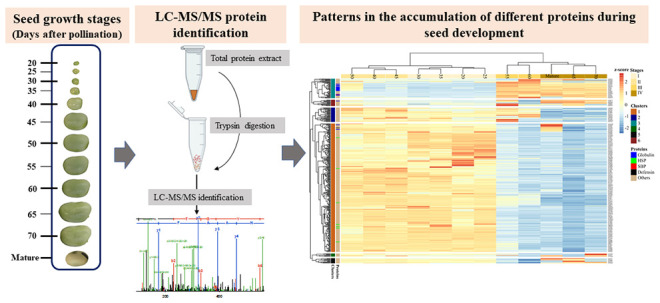

A major objective in faba bean breeding is to improve its protein quality by selecting cultivars with enhanced desirable physicochemical properties. However, the protein composition of the mature seed is determined by a series of biological processes occurring during seed growth. Thus, any attempt to explain the final seed composition must consider the dynamics of the seed proteome during seed development. Here, we investigated the proteomic profile of developing faba bean seeds across 12 growth stages from 20 days after pollination (DAP) to full maturity. We analyzed trypsin-digested total protein extracts from the seeds at different growth stages by liquid chromatography-tandem mass spectrometry (LC-MS/MS), identifying 1217 proteins. The functional clusters of these proteins showed that, in early growth stages, proteins related to cell growth, division, and metabolism were most abundant compared to seed storage proteins that began to accumulate from 45 DAP. Moreover, label-free quantification of the relative abundance of seed proteins, including important globulin proteins, revealed several distinct temporal accumulation trends among the protein classes. These results suggest that these proteins are regulated differently and require further understanding of the impact of the different environmental stresses occurring at different grain filling stages on the expression and accumulation of these seed storage proteins.

## Introduction

Faba bean (*Vicia faba*, L.) has one of the highest seed protein content among legumes.^1^ Therefore, it is considered a good candidate for meeting the increasing global demand for plant proteins. One of the current legume breeding objectives is improving the protein composition to suit quality requirements for human and animal nutrition.^2,3^ In faba bean, the predominant class of seed protein belongs to the globulin protein family, namely, legumin, vicilin, and convicilin.^3−5^ Each of these protein classes is encoded by several structural genes.^6−8^ From a nutritional point of view, legumins are of particular interest due to their higher content of sulfur-containing amino acids (S-AA) compared to other globulins.^3^ Therefore, cultivars of legumes with higher proportions of sulfur-rich proteins will be of great interest in human and animal nutrition. On the other hand, protein properties such as solubility, heat-induced gelation, foaming, and emulsifying capacity are essential for the food processing industry. However, although there is currently a lack of direct evidence in faba bean, studies in other species show that the accumulation of seed storage proteins is a complex biological process regulated by genes involved in transcription, synthesis, and transport.^9−11^ Thus, a better understanding of seed development and the dynamics of protein deposition is critical for the genetic manipulation of protein composition by conventional breeding, genetic engineering, or gene editing.

Temporal differences in the expression of major seed storage proteins during seed development have been reported in faba bean and other legume species. For instance, in developing faba bean seeds, De Pace et al.^12^ reported that the accumulation of certain vicilin protein bands preceded legumin by 4 days, while the legumin A-type bands were observed before those of legumin B-type. In addition, Panitz et al.^13^ showed that the cDNA of legumin B4 and vicilin had a diphasic accumulation pattern. These storage proteins started accumulating at 6 DAP and then disappeared before they started accumulating again at the cotyledon expansion stage. Differences in the timing of protein accumulation or gene expression for specific storage proteins during seed development have also been reported in other legumes, such as Medicago,^14,15^ pea,^16^ and soybean.^17^ From a nutritional standpoint, temporal differences in protein composition during seed development mean that changes in the environmental conditions at certain seed filling stages would modulate the protein composition and quality in the mature seeds. According to Henriet et al.,^18^ under sulfur-deficient growth conditions, the accumulation of legumin proteins was significantly reduced in mature pea seeds. This reduction was accompanied by downregulation of the expression of two legumin genes during seed development. In addition, in soybean, the abundance of several seed storage proteins was reported to vary considerably depending on whether genotypes were grown in the field or a glasshouse,^19^ indicating changes in protein composition due to prevailing conditions during seed growth. Therefore, better characterization of seed development and accumulation patterns of different storage proteins is the first step toward the rational modification of seed protein composition through genetics or agronomic practices.

Previous studies on the protein accumulation patterns in developing faba bean seeds development and were limited by the number of proteins studied and the sensitivity of the techniques used. For instance, De Pace et al.^12^ used one-dimensional sodium dodecyl sulfate-polyacrylamide gel electrophoresis (1D SDS-PAGE) to investigate the accumulation of few vicilin and legumin bands. On the other hand, Panitz et al.^13^ studied vicilin and legumin accumulation using a single cDNA for each protein. This study aimed to characterize faba bean seed development and the associated temporal trends in seed protein composition with particular emphasis on storage proteins belonging to legumin, vicilin, and convicilin. We used LC-MS/MS and label-free quantification to identify and quantify seed proteins across 12 seed developmental stages.

## Materials and Methods

### Plant Material and Growth Conditions

In this study, we used the inbred line Hedin/2, a spring faba bean line from Germany with a thousand seed weight of ∼500 g.^20^ This inbred line is of high genetic purity that has been used as a parent in multiple study populations.^21−23^ Also, this line has been used in faba bean genome sequencing project (https://projects.au.dk/fabagenome/).

Thirty single plants were grown in the glasshouse using 3 L pots containing homogeneously mixed compost (John Innes No. 2, Clover Peat, U.K.) at the Crop and Environment Laboratory (CEL) of the University of Reading, U.K. Plants were well-watered and received supplemental lighting to achieve 16 h of light per day using high-pressure sodium lamps providing a photosynthetic photon flux density of about 600 μmol m^–2^ s^–1^. The experiment was conducted during the winter season from November to February, with temperatures between 12 and 24 °C, on average.

At the flowering stage, individual flowers were tagged, and the date of pollination date was recorded on the day when the standard petal curved back and spots on the wing petals were visible without undue effort. Flowers were also tripped by hand during tagging to ensure high pod set rates. After successful fertilization, pods were sampled at 20, 25, 30, 35, 40, 45, 50, 55, 60, 65, and 70 DAP. Pods were carefully removed from plants; the number of the sampled node was recorded and then immediately frozen in liquid nitrogen and stored at −80 °C until further analysis. The mature seed sample was obtained from pods that naturally dried on the plants.

### Pod and Seed Development Measurements

At the end of the sampling process, pods belonging to each developmental stage were combined after visually examining any apparent off-types, such as pods with outlier sizes for the particular growth stage. Before removing seeds, pods were photographed along a 15 cm long ruler with a 1 mm scale. The same was done for the seeds before and after freeze-drying. Images were then processed with ImageJ software,^24^ where pod and seed lengths and seed size (based on the area of seeds laying on one of the cotyledons) were measured. Sample details are given in Table S1.

### Crude Protein Content Analysis

**Figure 1:**
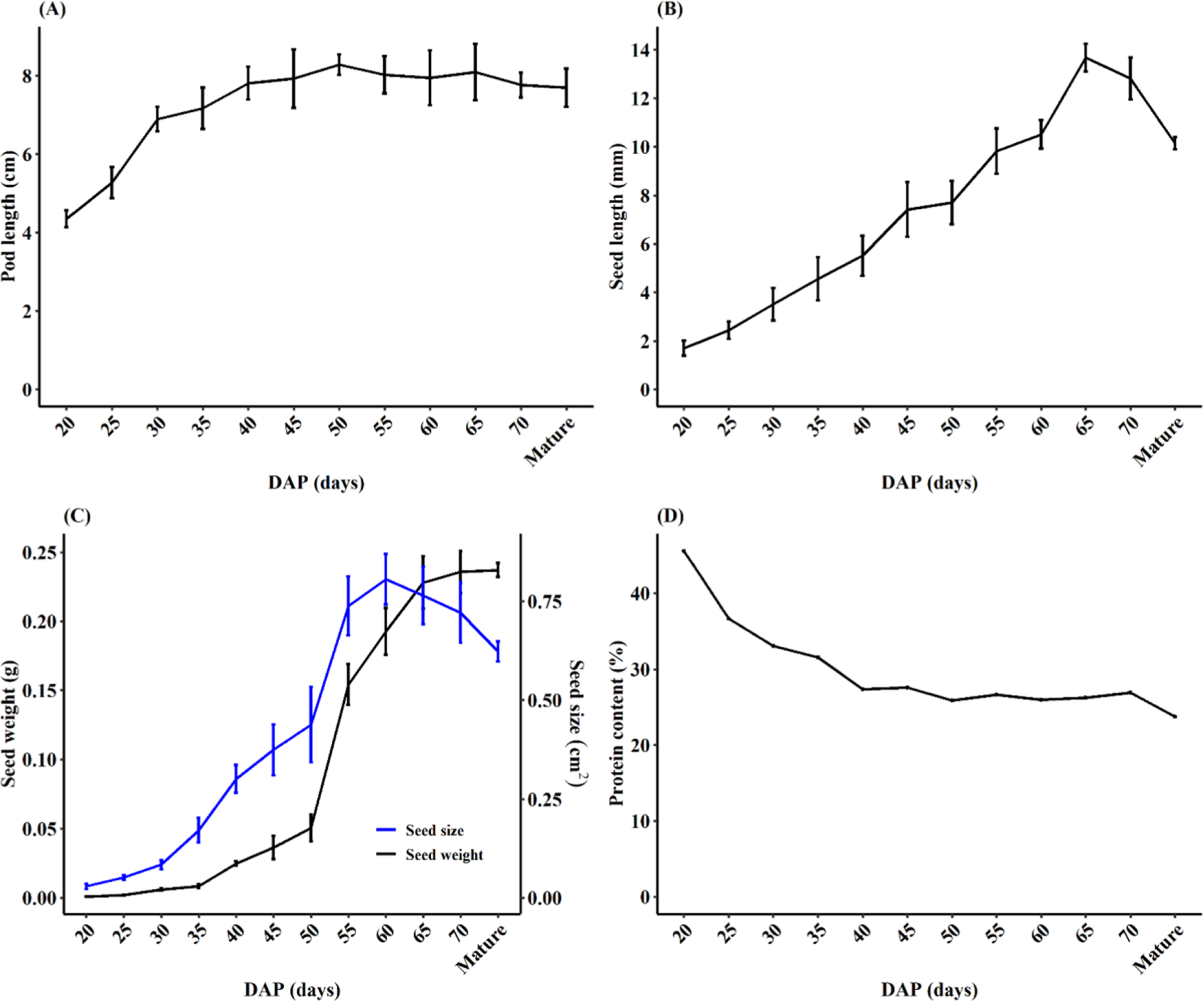
Characteristics of developing faba bean seeds. (A) Pod lengths between 20 days after pollination (DAP) as measured on 5–10 pods per growth stage. (B) Fresh seed length. (C) Weight and size of freeze-dried seeds. (D) Protein content on a dry weight basis for 12 growth stages. The error bars in (A–C) represent mean ± SD.

Seeds were freeze-dried (Super Modulyo, Edwards Vacuum, West Sussex, U.K.) until a constant weight was obtained (∼78 h) and then ground to homogeneous powder using a mortar and pestle. Then, depending on the sample availability, ∼60 to 100 mg of seed flour was analyzed in a LECO carbon/hydrogen/nitrogen determinator (628 Series, LECO). Subsequently, nitrogen content was converted to protein content using the 5.4 conversion factor.^25^ Due to the limited sample, the protein content at 20 DAP was not measured but imputed from the data points of all five traits described in Figure 1 using the mice R package.^26^

### Total Protein Extraction

Protein extraction was conducted as described by Scollo et al.^27^ Briefly, to remove phenolic compounds, a cold (∼4 °C) aqueous acetone (80% v/v) containing 5 mM sodium ascorbate was added to the samples at a 1:20 sample to buffer ratio. Then, the suspension was vortexed for 1 min and centrifuged at 1500*g* for 10 min at 4 °C. This step was done twice, and the supernatant was discarded each time. The sample was then washed with cold acetone and air-dried. Next, total proteins were extracted with a solution containing 7 M urea, 2 M thiourea, and 20 mM dithiothreitol (DTT). After shaking the suspension at 300 rpm for 1 h at room temperature, samples were centrifuged at 1500*g* for 15 min at 4 °C, and the supernatant was collected. The protein concentration of the samples was assessed with the Bradford method^28^ using a SpectraMax i3x microplate reader (Molecular Devices, U.K.). These samples were separated on NuPAGE 10% Bis–Tris precast SDS-PAGE gels as described by Warsame et al.^4^

### Trypsin Digestion

From the total protein extract for each developmental stage, technical triplicates containing ∼0.2 mg of protein were created. Then, 50 mM ammonium bicarbonate was added to each tube to bring the urea concentration to ∼1 M. Additional DTT was added to each tube at 10 mM final concentration and then was incubated for 30 min at 37 °C. Next, sulfhydryl groups were alkylated by the addition of iodoacetamide (IAA) at a final concentration of 20 mM. For protein digestion, trypsin (Promega, U.K.) was added at a 1:100 protease/protein ratio (w/w) and the solutions were incubated overnight at 37 °C. The reaction was terminated by freezing samples at −20 °C. To confirm that all proteins in the samples were completely digested, aliquots of the digested samples were loaded on 1D SDS-PAGE gel. Finally, samples were dried in a centrifugal vacuum concentrator. The mass spectrometry analysis was conducted as described by Warsame et al.,^4^ with each of the three technical replicates for each sample being analyzed three times. Thus, nine MS/MS data files were obtained for each developmental stage, except for 60 DAP that was analyzed in duplicate and had six data files.

### MS/MS Data Analysis

The raw MS/MS data were searched against the NCBI database using an in-house version of the MASCOT search engine (Matrix Science, U.K.) via Mascot Daemon with the file conversion performed using ProteoWizard. The MASCOT search parameters were set as described by Warsame et al.^4^ Then, to obtain a final list of protein accessions identified in each seed developmental stage, proteins identified in the technical replicates were merged, and the duplicated protein IDs were removed. For functional clustering of the identified proteins, protein sequences were functionally annotated using the MapMan4 framework and its associated online tool Mercator4.^29^ Considering the relatively large number of the resultant protein functional clusters obtained from MapMan4, we further grouped proteins into a few clusters according to Bevan et al.^30^

To assess the relative abundance of proteins across seed developmental stages, we conducted a label-free quantification using the MaxLFQ algorithm^31^ implemented in MaxQuant software.^32^ In this analysis, to reduce the computational power requirements, a protein database containing the proteins identified by MASCOT analysis was used as a reference for quantification. The details of the quantification parameters are given in Table S2. The downstream analysis, including normalization, imputation, and clustering, was conducted in Perseus software.^33^ Considering the differences in the number of proteins identified in each stage, we only used the proteins present in at least 7 of the 12 stages and 5 of the technical replicates for downstream analysis, including the imputation of missing data. The R package “pheatmap” (https://cran.r-project.org/web/packages/pheatmap) was used to visualize the normalized average relative abundances obtained from Perseus software.

We used MEGA X software^34^ to infer the identified globulin proteins’ evolutionary relationships using the neighbor-joining method with 10000 replicates and the “ggtree” R package^35^ to visualize the data.

## Results and Discussion

### Features of Developing Faba Bean Seeds

This study used the small-seeded reference inbred line Hedin/2, which carried flowers from nodes 8 to 27 on the main (and sole) stem with seven flowers per node on average. It is important to note that faba bean genotypes can vary significantly in the number of flowers per node, the number of pods, and seeds per pod.^36,37^ Therefore, aspects of the described developmental stages should be regarded as genotype- and environment-dependent. It is worth mentioning that this study does not investigate the very early stages of postfertilization development since collecting sufficient seed samples before 20 DAP was not feasible due to the tiny seed size of Hedin/2 (<2 mm) in the early growth stages.

To provide biological context to the changes in the proteomic profile of the developing seeds, we recorded some of the morphological and biochemical features of the developing seed and pod tissues between 20 DAP and full maturity (Figure 1). The early growth phase is marked by the rapid growth of pods, which nearly doubled in length between 20 and 35 DAP and followed by more gradual growth to reach the maximum length at 50 DAP (Figure 1A). This early rapid pod development before the onset of seed filling has also been observed in soybean^38^ and serves to give ample space for the seed to develop. In contrast, seeds gradually increased in length almost linearly until 65 DAP (Figure 1B), which usually starts to lose water and shrink at maturity. On a dry seed basis, seed growth was characterized by a gradual increase in weight and area until 50 DAP and then a rapid increase in both parameters between 50 and 60 DAP (Figure 1C). At 60 DAP, the seed area peaked while dry weight increased substantially to reach its maximum at maturity (Figure 1C).

On the other hand, the percent crude protein content on a dry weight basis was the highest during the early stages of seed development but dropped rapidly from >40% to <28% by 40 DAP, remaining in the 26–28% range throughout the later seed developmental stages period (Figure 1D). A similar protein content pattern was reported in developing seeds of mung bean^39^ and soybean.^40^ The observed higher protein content (i.e., nitrogen content) in the early developmental stages is probably due to the higher concentration of structural proteins, enzymes, free amino acids, and other nitrogen-containing compounds associated with rapid cell division during these early stages. Also, at the early growth stages, other bulk seed components, chiefly starch, which normally dilutes protein content in the final stages, may not yet have accumulated in the seeds. In the mature seeds, however, the 24% protein content was comparable to the average protein content of Hedin/2 plants grown in the field (data not shown).

### 1-D SDS-PAGE Protein Profile of the Seed Developmental Stages

**Figure 2:**
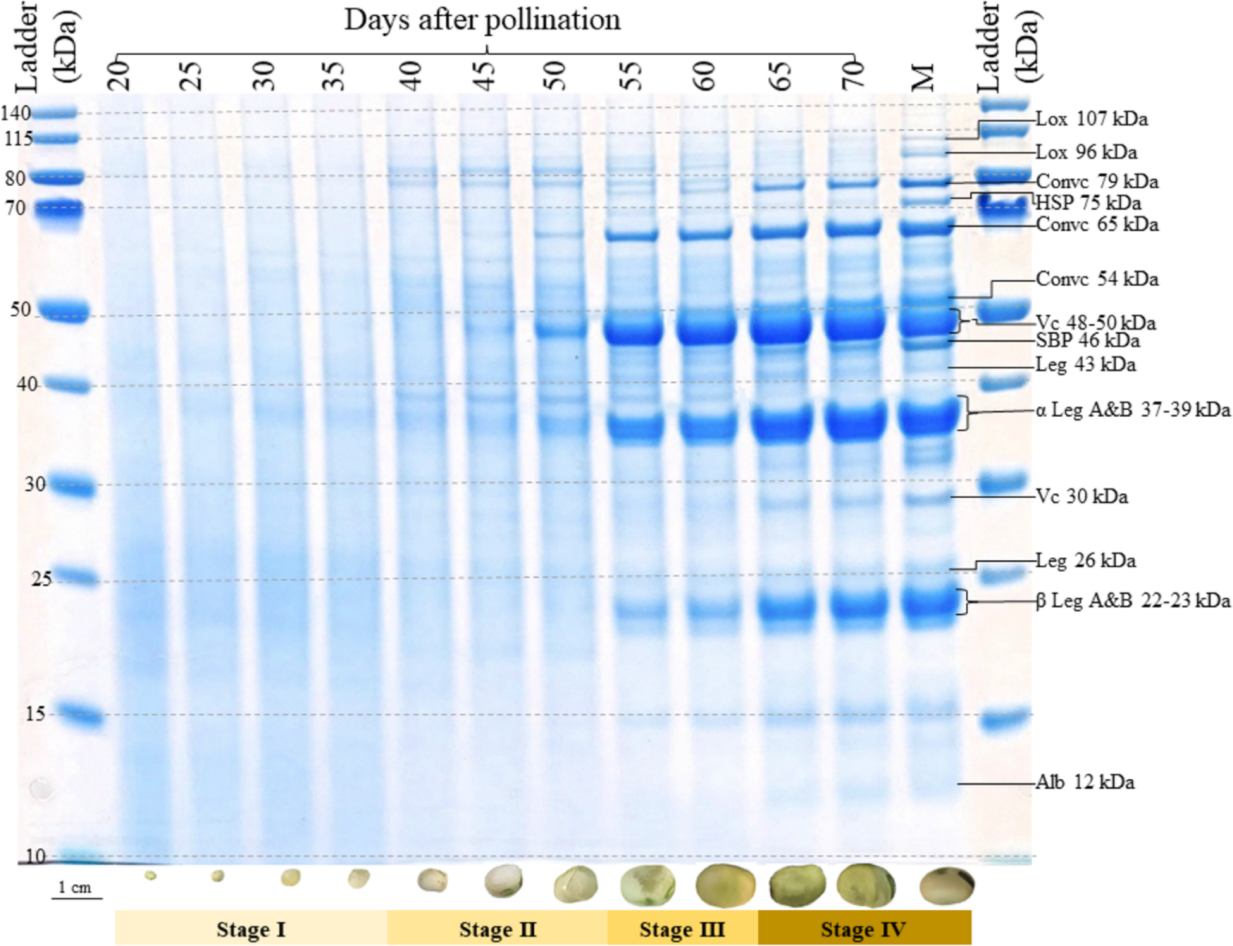
1D SDS-PAGE gel showing the protein profile of faba bean seeds harvested at 12 developmental stages. Molecular weights of individual bands are estimated with respect to the bands of the MW ladder in the leftmost lane, with sizes of the marker bands given in kDa. Abbreviated names of discrete and most abundant protein bands are based on mass spectroscopic identification of proteins in Warsame et al.^4^ and are listed in Table S5. At the bottom of each lane, sample images of freeze-dried seeds belonging to the pool representing each growth stage are shown; colored bars along the bottom of the gel denote the observed main phases of protein accumulation. A 1 cm scale bar for seed images is given on the bottom left.

The seed protein profile on a 1-D SDS-PAGE gel shows a clear timeline of the accumulation of different seed proteins across the 12 growth stages (Figure 2). The 20–35 DAP period is characterized by a greater apparent abundance of lower molecular weight proteins (<40 kDa) with no visible accumulation of major storage proteins. This period coincides with the stage of rapid seed and pod growth in Figure 1 and is denoted as stage I in Figure 2. From 40 to 50 DAP, seeds transition to the protein accumulation phase (stage II), and some seed storage protein subunits, including convicilin 65 kDa, vicilin 48–50 kDa, and α-legumin A&B 37–39 kDa, are visible by 50 DAP. At 55–60 DAP (stage III), vicilin 48–50 kDa appears to be more abundant than legumins, which is consistent with findings of De Pace et al.,^12^ who reported an earlier accumulation of vicilin bands compared to legumins. Finally, seeds enter a phase of accelerated protein accumulation at 65 DAP throughout which the protein profile is quite comparable to that of the mature seed (Figure 2).

### Functional Categories of Proteins Identified across Seed Developmental Stages

A total of 1217 proteins were identified by mass spectrometry analysis in the seed samples from 20 DAP to mature seeds (Table S4). This list included 36 of 104 proteins previously identified in protein bands of mature seeds of three different faba bean accessions.^4^ This relatively lower overlap between the two experiments can be attributed to differences in the sample characteristics of the two experiments. The LC-MS analysis of individual protein bands from 1D SDS-PAGE gel slices by Warsame et al.^4^ is more sensitive in detecting low abundant proteins compared to the whole proteome analysis conducted in this study, where the major storage proteins can mask the less abundant protein species. Traditionally, to identify the maximum absolute number of protein species in the total protein extracts from highly complex samples, a fractionation or depletion of the abundant proteins is performed before LC-MS analysis.^27^ However, since this study aimed to compare the unaltered relative abundances of seed proteins across seed developmental stages with the main interest in the dynamics of the most abundant storage proteins, fractionation or depletion was not considered appropriate. Also, considering the apparent gradient in the protein profile shown in Figure 2, fractionation of the samples would have produced vastly different outcomes in different samples. Compared with other studies on seed proteomics, where the more sensitive nanoflow LC-MS/MS was used, 1168 proteins were identified in mature barley seeds^41^ and 704 in total protein extract of cocoa beans.^27^ This variation underlines the variability in the number of proteins detected among species, which may be affected by sample preparation methods or settings used for the LC-MS analysis.

**Figure 3:**
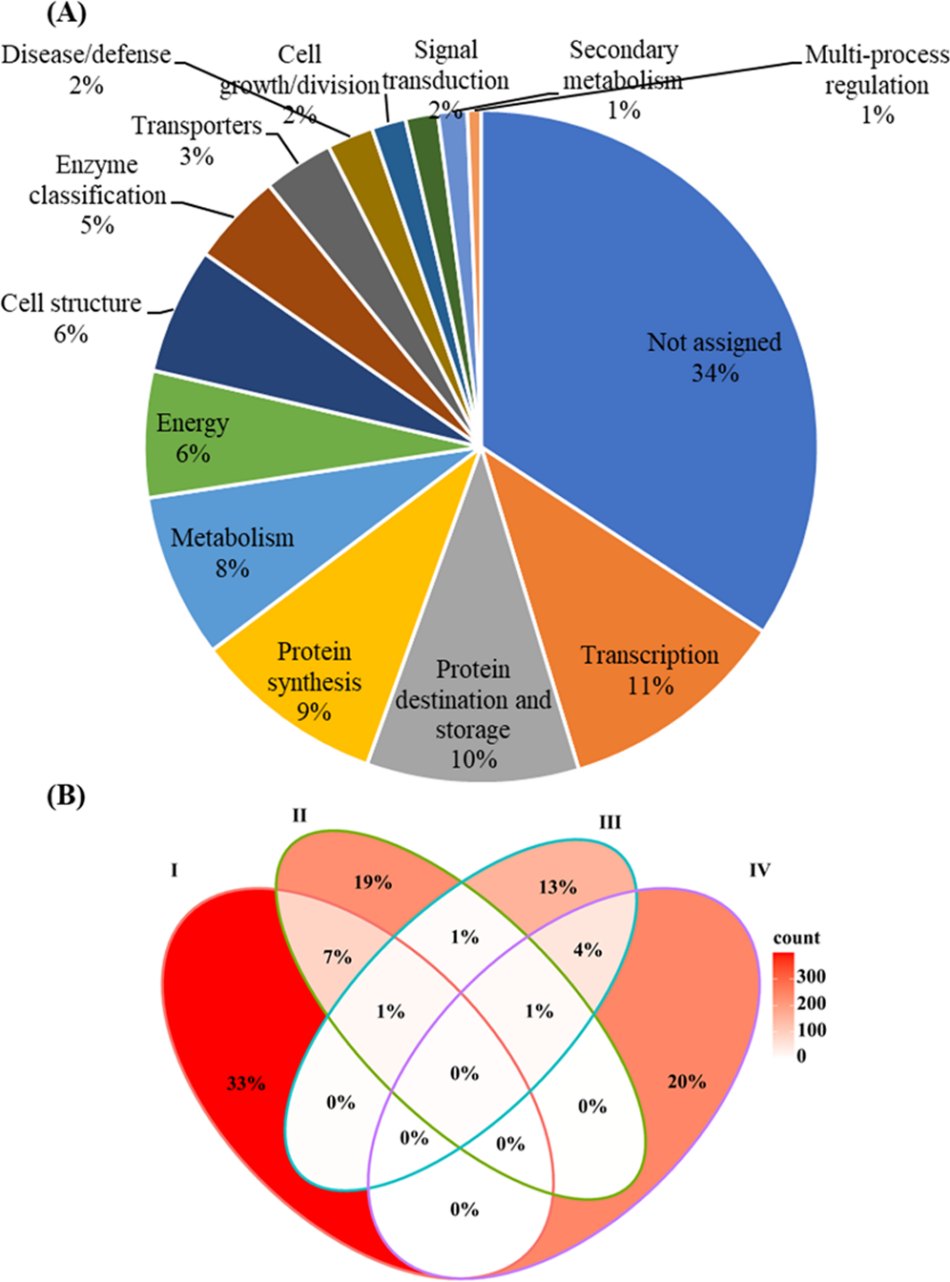
(A) Functional categories of the total list of 1217 proteins identified in 12 faba bean seed growth stages from 20 DAP to the mature stage. (B) Venn diagram showing the percentage of the total number of identified proteins that is specific or common among four developmental stages: I (20–35 DAP), II (40–50 DAP), III (55–60 DAP), and IV (65 to full maturity). The heat scale indicates the strength of overlap (red = high and white = low).

Proteins identified across the developmental stages could be assigned to 14 functional categories (Figure 3A) using MapMan4, which is a framework specifically designed for plant protein classification.^29^ After further grouping of the protein clusters, according to Bevan et al.,^30^ 34% of the protein sequences could not be assigned to any protein category, while the three largest groups, totaling 30% of all proteins identified, were proteins functioning in transcription, protein destination, and storage, and protein synthesis at 11, 10, and 9%, respectively. These numbers are consistent with the nature of the developing seed, particularly the cotyledon, as a storage organ undertaking active protein synthesis and deposition.

Looking at the developmental stage specificity of the identified proteins, we found that the overall overlap in the protein profile was strikingly low, and the majority of proteins identified at each stage were found only in that stage. For instance, there were 33, 19, 13, and 20% of the proteins specific to stages I (20–35 DAP), II (40–50 DAP), III (55–60 DAP), and IV (65 to full maturity), respectively (Figure 3B). The far greater proportion of the proteins identified in the early stages (ca. 33%) compared to later stages is consistent with the SDS-PAGE patterns in Figure 2. This figure showed a transition from a protein smear caused by many proteins of every possible size at 20 DAP to a clear banding pattern featuring a small number of very abundant protein species. The maximum number of proteins in common between different stages was 7%, shared between stages I and II. This is expected due to shifts in seed proteome across the seed life cycle from actively dividing cells at early growth stages through nutrient (mainly starch and protein) accumulation phase and, eventually, desiccation and dormancy at maturity.^42^

**Figure 4:**
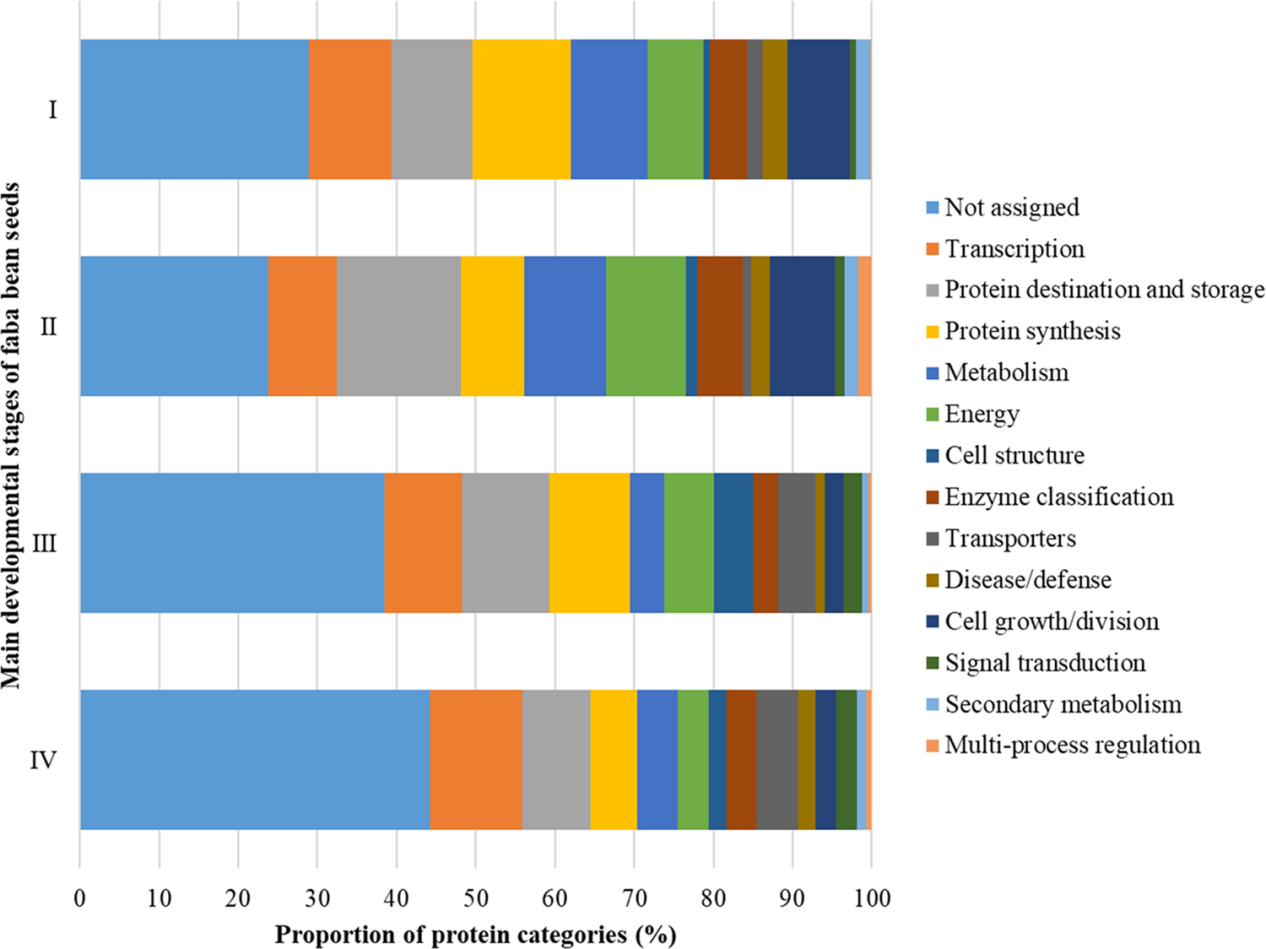
Changes in the functional categories of faba bean seed proteins through four major developmental stages.

Growth stages also differed in the dominant protein functional classes. Interestingly, although the major storage proteins did not accumulate significantly before 45 DAP (Figure 2), there was a high relative abundance of proteins involved in protein synthesis and protein destination and storage in the early stages (Figure 4). These earlier stages also had higher percentages of proteins involved in and energy production pathways (Figure 4), which agreed with expression patterns reported in soybean seed proteins^38^ and their gene cDNAs.^17^ It is worth noting that some of the differences in the representation of some protein clusters at certain growth stages could be due to underlying sample properties, particularly in the later developmental stages. At stages III and IV, storage proteins are overwhelmingly abundant, and our method may not have detected the less abundant protein classes.

### Temporal Trends in the Relative Abundance of Seed Proteins during Seed Development

As shown in Figure 2, individual proteins could vary progressively in abundance across seed growth and development stages. Thus, label-free quantification was performed to quantify these trends, resulting in calculating the relative abundances of 344 proteins across the developmental time course. Generally, accurate quantification of hundreds or thousands of proteins in a sample is a major challenge in proteomic studies. Each quantification method has its limitations and advantages in terms of cost, sample preparation requirements, and sensitivity.^43^ Here, considering the relative simplicity of the approach taken, the number of proteins identified and quantified was considered more than satisfactory. Additionally, as mass spectrometry is becoming the gold standard method for proteomic works, standard-flow LC-MS/MS has been regarded as a potential alternative to the more sensitive but expensive nano-LC-MS/MS systems.^44,45^

**Figure 5:**
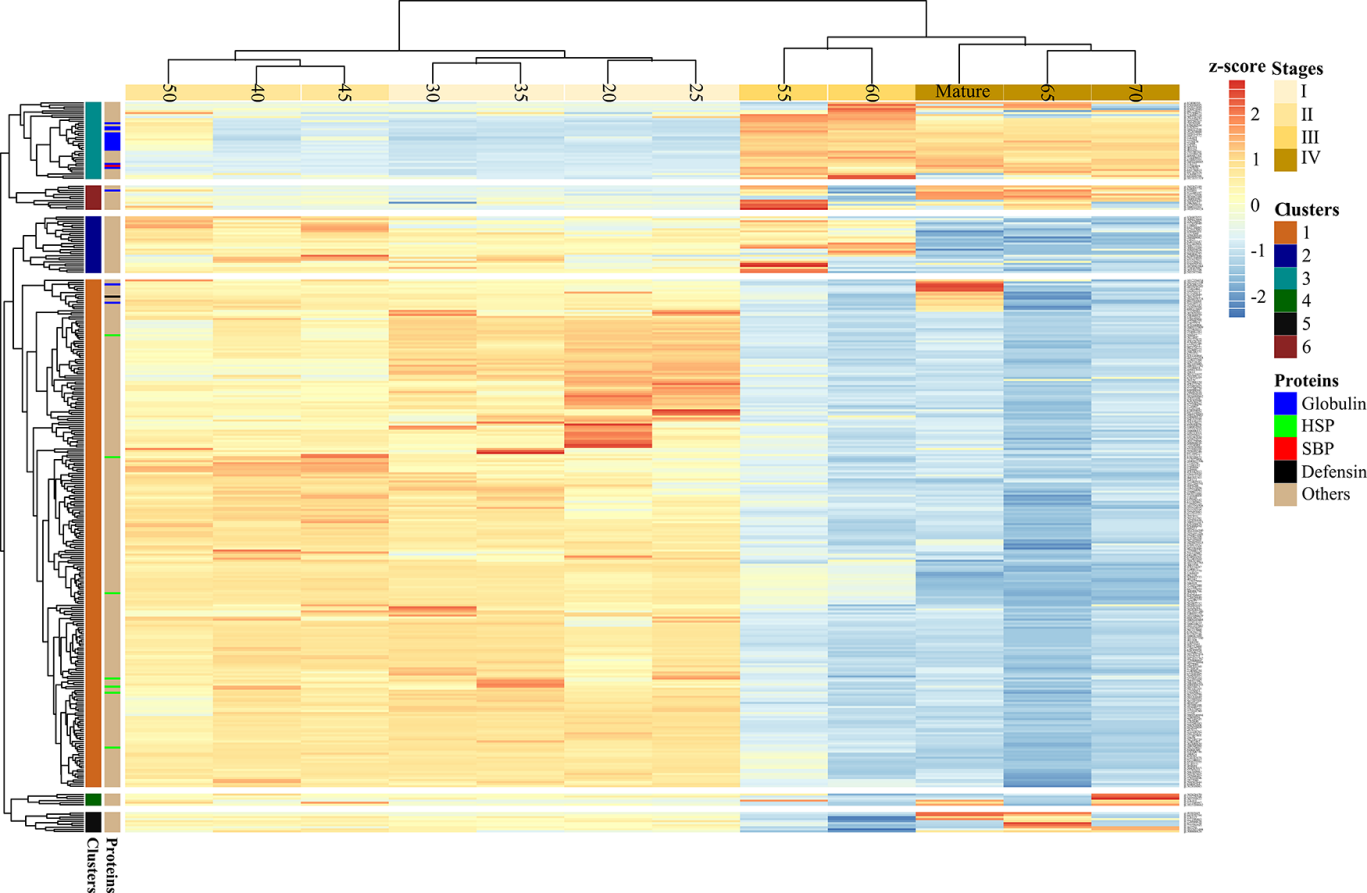
Heatmap showing the relative abundances of 344 proteins between 20 DAP to maturity. The four major developmental phases are indicated by gradient colors ranging from light to dark yellow. Color-coded protein families are globulins (including legumins, vicilins, and convicilins), heat shock proteins (HSPs), sucrose-binding protein (SBP), defensin, and others.

Quantified proteins showed characteristic differences in their relative abundance patterns across seed developmental stages and could be clustered accordingly into six groups (Figure 5). For simplicity, the proteins will be referred to by their generic names or accession numbers, as they are not yet adequately annotated in faba bean. Details of the quantified 344 proteins are summarized in Table S5.

At the early growth stages (20–35 DAP), proteins with the highest relative abundance were nonstorage proteins (cluster 1), including those involved in transcription like histones H2A and H2B (gi|593699727 and gi|470116864, respectively) and energy production, such as glyceraldehyde-3-phosphate dehydrogenase (gi|462138). Also, among seven chaperones identified in this analysis, the heat shock protein (gi|473217) was consistently upregulated until 45 DAP. This protein is 87% identical to the other HSP71.2 (gi|562006), which was previously found abundant in the dry, mature faba bean seeds.^4^ In pea, isoforms of these HSP proteins (PsHSP71.2, PsHSC71.0, and PsHSP70b) were identified in different plant tissues and considered to play distinct biological functions related to repairing and preventing cell damage and acquiring adaptive thermotolerance.^46^

As seeds developed, cluster 2 proteins reached their highest intensity, mainly between 45 and 60 DAP. Though the majority of proteins in this group were not functionally annotated, the group included pectin acetylesterase 8-like (gi|356558882), annexin-like (gi|459649445, gi|828335547), and putative copper-transporting ATPase 3 (gi|734387082). On the other hand, proteins in cluster 3 mainly consisted of seed storage proteins, which started to accumulate around 45 DAP and continued steadily until maturity. Other nonstorage proteins which were differentially accumulated during late grain filling included a sodium/hydrogen exchanger (gi|29539109). This protein has been reported to play a critical role in protein trafficking and the biogenesis of protein storage vacuoles (PSV) in *Arabidopsis*.^47^ Furthermore, a putative sugar phosphate transporter (gi|302854600), lectin–glucose complex (gi|82408030), and other proteins with protein maintenance functions (gi|971508673) were in high relative abundance concomitantly to the phase of high protein accumulation. The remaining clusters (4–6) contained fewer proteins with distinct accumulation patterns. For instance, proteins in cluster 5 were upregulated mainly at late stages (65 DAP to maturity) and included proteins that may function in defense and stress adaptation, such as ascorbate peroxidase (gi|731359393) and mitochondrial chaperonin (gi|461736). Another cluster 5 member, a serine protease inhibitor (gi|308800626), which is part of a gene family that regulates endogenous proteolysis in seeds and cell death during plant development and senescence,^48^ was also upregulated at 70 DAP and maturity.

### Diversity in the Accumulation Patterns among Major Storage Proteins

**Figure 6:**
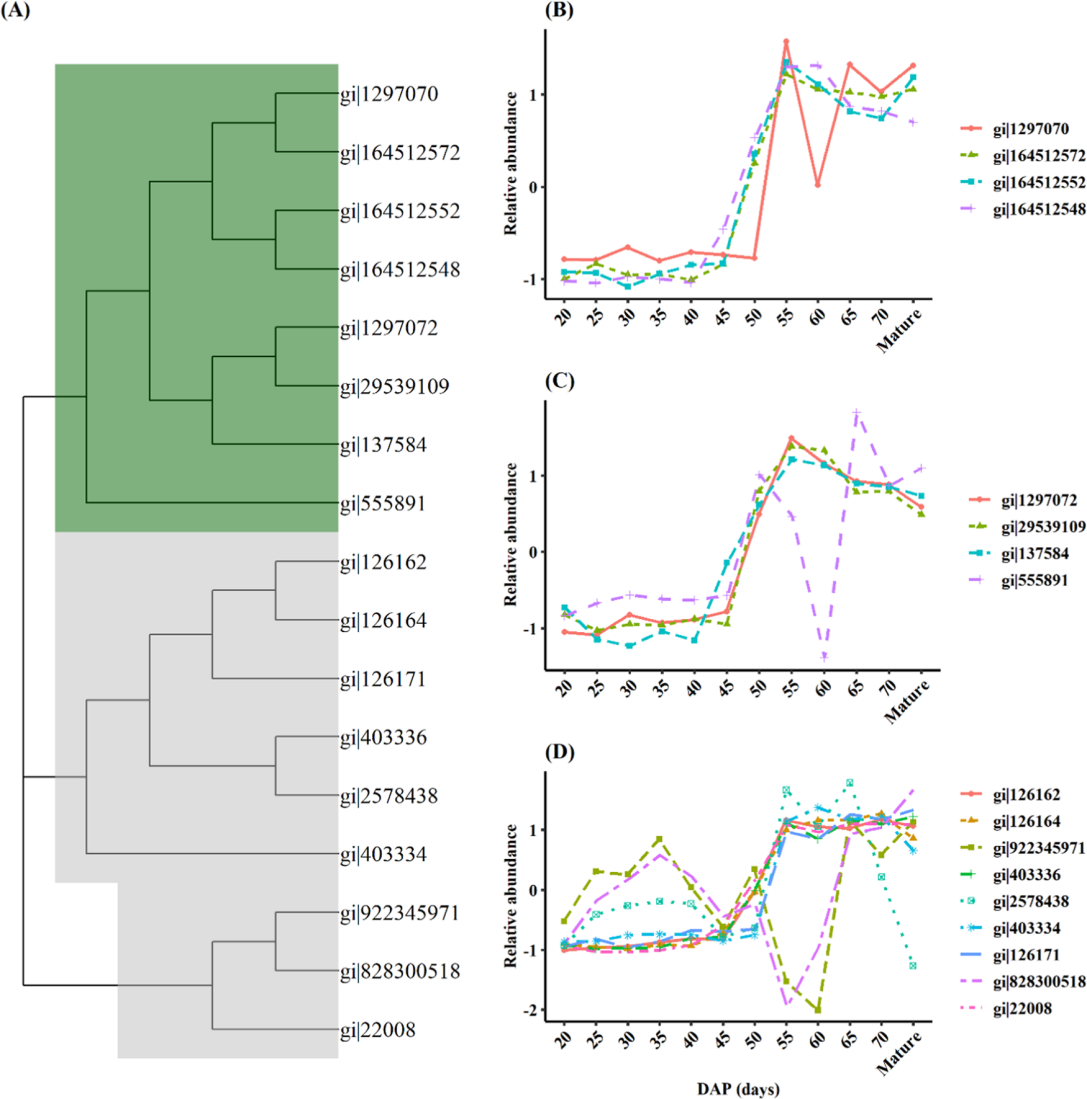
Patterns in the relative abundances of 17 globulin proteins across 12 seed growth stages between 20 DAP to maturity. These proteins are a subset of cluster 3 described in Figure 5. (A) Phylogenetic tree showing relationships between the different globulin genes, where vicilin/convicilins nodes are highlighted with dark green and legumins with gray color (B–D).

As previously noted, the globulins were primarily (though not exclusively) found in cluster 3 (Figure 5), characterized by a high accumulation in the late stages of grain filling. However, subtle differences in the onset and peak time of an individual protein could be highly significant given the very high absolute abundance of these storage proteins. Therefore, we compared the relative abundances of 17 globulins, including convicilins, vicilins, and nine legumins, to reveal the differences in the accumulation patterns within and between these protein classes (Figure 6).

Convicilins and vicilins are collectively called 7S globulins, with convicilin being considered a subunit of vicilin.^49,50^ This hypothesis is supported by their phylogenetic relationship, where they form a separate cluster (Figure 6A). In general, proteins belonging to this globulin group had similar accumulation patterns. They started to accumulate from 45 DAP and reached their maximum abundance between 55 and 60 DAP (Figure 6B,C), after which their relative abundance declined gradually. However, at 60 DAP, one convicilin (gi|1297070) and a vicilin-like protein (gi|555891) were significantly reduced, with the latter being phylogenetically distant from the other 7S globulins (Figure 6A). The earlier accumulating vicilin (gi|137584) was also the most abundant in major protein bands of 48–50 kDa in the mature faba bean seeds.^4^ Based on its apparent molecular weight, this protein is likely to be the vicilin band reported to be synthesized 4 days earlier than legumins by De Pace et al.^12^

On the other hand, legumin proteins were more diverse in their accumulation patterns (Figure 6D). Of the nine legumin-type proteins, 6 had similar accumulation trends, where they started to deposit between 45 and 50 DAP and remained unchanged until maturity (Figure 6D). A biphasic accumulation pattern characterized the other three legumin-type proteins; they had higher relative abundance during early growth stages (25–45 DAP) but downregulated differently in the later growth stages. In particular, the sharp downregulation of two legumins (gi|922345971 and gi|828300518) between 45 and 60 DAP is intriguing and difficult to explain. A possible reason could be that the transcription of these genes could be sensitive to the prevailing cell conditions at this period. For instance, it has been reported that high sucrose content in the cell reduces the stability of legumin mRNA, leading to significantly reduced legumin protein wrinkled peas.^51^ However, this needs further investigation.

Nonetheless, the different temporal trends in the relative abundance of these globulin proteins confirm and expand the current knowledge on the accumulation of globulins in faba bean and other legumes. As was briefly mentioned in the Introduction section, De Pace et al.^12^ reported that legumin A subunits appeared to accumulate 2 days before the legumin B-type. In pea, gene expression analysis of developing seeds showed that legumin B-type gene (Psat3g055960) is highly expressed early (16 DAP) compared to legumin A-type (Psat3g058800), which had a maximum expression at 19–23 DAP.^16^ Similarly, the expression pattern of legumin K in *Medicago truncatula* indicated that it was synthesized earlier (∼16 DAP) compared to legumin A (24 DAP).^15^ Overall, the observed diversity in the timing of expression within the classes of storage proteins may be indicative of a complex regulatory system. Thus, we need a better understanding of the genetic basis controlling its fine-tuning and how it impacts the nutritional quality of the seeds.

In conclusion, the different accumulation patterns within and among seed protein classes found in this study suggests that a corresponding diversity may exist in transcriptional, translational, and post-translational regulation of faba bean seed protein expression and accumulation. This opens new avenues for further investigations into the identification of master regulatory factors driving the developmental switch from cell division and growth to protein deposition. For instance, the characterization of promoter sequences that may mediate differential responses to these master regulators can aid in selecting the best targets for genetic improvement of nutritional composition in faba bean. As, by far, the most comprehensive survey of faba bean seed protein accumulation patterns, the results of this study can contribute to the annotation of the currently in production Hedin/2 genome assembly. The availability of a complete reference genome sequence accompanied by detailed expression data will, in turn, permit the identification of important regulatory motifs of seed proteins.

## Supporting Information Available

Details of developing *V. faba* seed samples used for proteomic analysis (Table S1); summary of the parameter settings used in MaxQuant software for label-free quantification of relative protein abundances (Table S2); details of seed protein band labels in Figure 2 that were previously identified by LC-MS (Warsame et al.^4^) (Table S3); combined list of all 1217 proteins identified across four major growth stages (Table S4); list of the 344 proteins quantified by MaxQuant across 12 developmental stages of *V. faba* seeds (Table S5) (XLSX)

## Notes

^§^ Department of Biochemistry and Metabolism, John Innes Centre, Norwich Research Park, Norwich NR4 7UH, U.K.

## Abbreviations

DAP: days after pollination
1D SD-PAGE: one-dimensional sodium dodecyl sulfate-polyacrylamide gel electrophoresis
kDa: kilo Dalton
MW: molecular weight
MS: mass spectrometry
LC: liquid chromatography
